# Real‐World Adherence to Repeat Colorectal Cancer Screening With the Multi‐Target Stool DNA Test in a Large, Insured, and Average‐Risk Population

**DOI:** 10.1002/cam4.71314

**Published:** 2025-10-29

**Authors:** Mallik Greene, Joseph Anderson, Joseph LeMaster, Jeffrey Arroyo, Jorge Zapatier, Jemel Bingham, Raja Kakuturu, Jordan J. Karlitz, Quang Le

**Affiliations:** ^1^ Exact Sciences Corporation Madison Wisconsin USA; ^2^ Geisel School of Medicine Dartmouth College Hanover New Hampshire USA; ^3^ University of Kansas School of Medicine Kansas City Kansas USA; ^4^ AltaMed Health Services Los Angeles California USA; ^5^ Digestive Diseases Consultants Altamonte Springs Florida USA; ^6^ Hartford Healthcare Medical Group Hartford Connecticut USA

**Keywords:** adherence, colorectal cancer, digital outreach, multi‐target stool DNA test, repeat screening, stool‐based screening

## Abstract

**Introduction:**

Guidelines recommend starting average‐risk colorectal cancer (CRC) screening at age 45 years, and thereafter repeating screening tests at regular intervals. However, US screening rates are currently suboptimal. This study evaluated adherence to repeat CRC screening with the non‐invasive multi‐target stool DNA (mt‐sDNA) test among US adults.

**Methods:**

This was a retrospective claims‐based analysis of individuals aged 45–75 years at average risk for CRC who had previously completed the mt‐sDNA test. Individuals received the repeat mt‐sDNA kit based on a point‐of‐care order. The primary outcome was adherence, defined as the return of a kit with a valid result within 1 year. Secondary outcomes included rates of follow‐up colonoscopy.

**Results:**

The analysis included 352,253 patients. Most patients were female (62.1%), White (60.9%), had no comorbidities (76.5%), and underwent one earlier mt‐sDNA test (98.1%). Overall adherence for repeat screening was 86.2%. In logistic regression analysis, higher household income ($100,000–$200,000: 2.05; 1.49–2.77; > $200,000: 2.46; 1.75–3.40; both vs. < $25,000) and receipt of digital outreach (1.36; 1.33–1.40 vs. no digital communications) were associated with higher adherence to repeat screening. Mean (95% CI) time to a successful result was 35.6 (35.4–35.7) days. Among patients who tested positive, 75.8% underwent colonoscopy.

**Conclusions:**

In this large real‐world study, adherence to repeat screening with the mt‐sDNA test was high. These findings underscore the effectiveness of the mt‐sDNA test in improving CRC screening adherence.

## 1 Introduction

Colorectal cancer (CRC) is the third most commonly diagnosed cancer and the second most common cause of cancer‐related deaths in the United States, accounting for an estimated 153,020 new cancer cases and 52,550 deaths in 2023 [1]. Screening for CRC can detect early‐stage disease and precursor lesions, which allows for earlier treatment and consequently improves prognosis and reduces CRC‐related morbidity and mortality [2, 3, 4].

The US Preventive Services Task Force (USPSTF) recommends CRC screening for all individuals aged 45–75 years who do not have signs or symptoms of CRC and are at average risk for CRC, while selective screening is recommended for those aged 76–85 years [2]. Several screening methods for CRC are currently available in the United States. Direct visualization tests are invasive procedures and include colonoscopy and flexible sigmoidoscopy [2, 5]. By contrast, stool‐based tests are relatively quicker to perform, non‐invasive, and can be performed at home; this test type includes the guaiac fecal occult blood test (gFOBT), the fecal immunochemical test (FIT), and the multi‐target stool DNA (mt‐sDNA) test [2]. Positive results from stool‐based screening tests need to be followed up with a timely colonoscopy for definitive diagnosis [6].

In a national effort to accelerate CRC screening progress, the National Colorectal Cancer Roundtable (NCCRT) established a goal to reach CRC screening rates of 80% and higher in age‐eligible adults in every community across the United States [7, 8]. However, current CRC screening rates are suboptimal, with the US Department of Health and Human Services estimating CRC screening rates among adults aged ≥ 45 years at only 63.5% in 2023 [9].

The mt‐sDNA test is a non‐invasive option for average‐risk CRC screening that detects both fecal hemoglobin and human DNA biomarkers, in contrast with the gFOBT and FIT tests, which detect only blood components in stool samples (heme and hemoglobin, respectively) [10, 11]. The mt‐sDNA test was initially approved by the US Food and Drug Administration in 2014 for individuals aged ≥ 50 years, with the approved indication expanding in 2019 to include individuals aged 45–49 years [12, 13]. The expanded indication is in line with USPSTF and American Cancer Society (ACS) guidelines, which now recommend the screening of adults aged ≥ 45 years with average risk of CRC [2, 6].

Persistent adherence refers to adherence (completion of prescribed tests) with repeated screening tests over a period of time and is key to the efficacy and effectiveness of CRC screening programs [5]. Consistent adherence is particularly critical for stool‐based screening, where efficacy is dependent on patient commitment and adherence to multiple rounds of testing [2, 5]. Recommended intervals for repeat CRC screening depend on the type of screening tests used [5]. For stool‐based tests, screening intervals range from annually for the gFOBT and FIT to every 3 years for the mt‐sDNA test [2, 6].

A recent study evaluating adherence to mt‐sDNA testing showed that the overall adherence rate was 71.3% among first‐time users who were covered by commercial insurance, Medicare, or Medicaid in the United States [14]. There is a need for real‐world evidence on the rates of repeat screening with the mt‐sDNA test among individuals aged ≥ 45 years. Hence, the objective of this real‐world study was to evaluate adherence to repeat CRC screening with the mt‐sDNA test and the rate of follow‐up colonoscopy in the event of a positive mt‐sDNA test among average‐risk individuals aged 45–75 years across different health insurance types and demographic subgroups in the United States.

## 2 Materials and Methods

### 2.1 Data Sources

This was a retrospective study using data sourced from mt‐sDNA records linked to the Komodo Health Claims database from January 1, 2017, to December 1, 2023. The Komodo Health Claims database is a comprehensive, longitudinal dataset built from real‐world U.S. healthcare encounters, covering over 330 million patient lives. It includes fully adjudicated medical and pharmacy claims data from a wide range of payers, including commercial, Medicare Advantage, and Medicaid plans (though it lacks coverage of uninsured patients). Its depth and breadth make it particularly valuable for real‐world evidence studies. Records for aggregated laboratory data for mt‐sDNA screening were obtained from Exact Sciences Laboratories LLC (ESL; Madison, WI), the exclusive national laboratory for mt‐sDNA (Cologuard) testing. The ESL database includes information on all mt‐sDNA laboratory data, such as data on laboratory test results, patient outreach, payor, and basic demographics. The link between the national claims database and ESL data through tokenization created integrated data that provided complete information on mt‐sDNA testing and results from ESL, as well as comprehensive health information derived from the claims database. US census data were used for ZIP code median household income data.

All data were de‐identified and compliant with the Health Insurance Portability and Accountability Act (HIPAA). The study was considered exempt research under 45 CFR § 46.104(d)(4) as it involved only the secondary use of data that were de‐identified in compliance with HIPAA, specifically, 45 CFR § 164.514.

### 2.2 Study Design and Population

The study included individuals in the United States aged 45–75 years who were at average risk for CRC, had a previous mt‐sDNA test with a negative result, were undergoing mt‐sDNA repeat screening, and received an mt‐sDNA test kit from ESL based on a point‐of‐care order that was shipped between January 1, 2017, and December 1, 2023. Eligible patients also had health insurance plan coverage (commercial, managed care organization [MCO], Medicare, or Medicare Advantage), and were continuously enrolled in a health plan with medical claims at least one year before and after an mt‐sDNA repeat screen order. The index date was defined as the shipment date of the mt‐sDNA test within an index year (2017–2023).

Individuals were excluded if they were aged < 45 or > 75 years; had ESL records that were not linked with the claims database; were considered high risk for CRC; resided outside of the United States; or had missing information regarding their age, sex, race/ethnicity, Charlson Comorbidity Index (CCI) score, urban/rural classification, or prescribing provider specialty.

Average risk for CRC was defined as having no prior diagnosis indicating high risk for CRC, including diagnosis of CRC, inflammatory bowel disease or ulcerative colitis, familial polyposis syndromes, colonic adenomas, or colonic polyps. Individuals also had no documented family history of CRC or prior colectomy. The conditions used to determine average‐risk status for CRC were identified using International Classification of Diseases, Tenth Revision diagnosis codes documented in the linked medical claims data. Family history of CRC was also identified using relevant diagnosis codes where available. These diagnoses were assessed during the 12‐month baseline period prior to the index mt‐sDNA test order. We used linked claims data to ensure the consistent application of the average‐risk inclusion criteria rather than relying on a separate algorithm beyond claims‐based diagnosis code identification.

All individuals for whom the mt‐sDNA test was ordered were offered an enhanced navigation and support system that included automated and live phone calls, mailed letters, emails, and short message service (SMS) messages, which aimed to maximize screening adherence. The mt‐sDNA kit included educational materials that outlined the steps involved in the CRC screening process and encouraged patients to discuss a follow‐up colonoscopy (colonoscopy recommended based on a positive mt‐sDNA test result) with their healthcare provider in the event of a positive test result. Individuals' opt‐in preferences were communicated to ESL from their provider. Based on each individual's preference, digital outreach (SMS + email, SMS only, or email only) or no digital outreach communications were assigned.

### 2.3 Outcome Measures

The primary study outcome was the rate of adherence to repeat screening with the mt‐sDNA test, defined as the return of a kit that resulted in a valid test result (negative or positive) within 1 year from the index (shipment) date. Secondary outcomes included: (i) time to adherence, defined as the number of days between the date of mt‐sDNA kit shipment to the participant and the date of a valid test result, and (ii) the rates of positive mt‐sDNA test results and follow‐up colonoscopies (assessed 12 months after a positive mt‐sDNA test).

### 2.4 Statistical Analysis

Descriptive statistics were used to summarize the baseline characteristics of the study population, adherence to repeat screening with the mt‐sDNA test, days to a successful test result, the rate of positive mt‐sDNA test results, and the rate of follow‐up colonoscopies. Baseline characteristics, adherence rates, and days to a successful result were described for the overall cohort and by health insurance plan, and compared within categories of characteristics including age group, sex, race/ethnicity, CCI score, urban/rural classification, median household income by patient ZIP code (obtained from US census data), specialty of the prescribing provider, receipt of digital outreach, and number of earlier mt‐sDNA tests. Because the claims database does not distinguish between Hispanic and non‐Hispanic subgroups within each racial category, we were unable to separately classify patients as, for example, non‐Hispanic Black or non‐Hispanic White. Adherence results were stratified by health insurance type to provide a more granular view of screening behaviors across different segments of the insured population.

Frequencies and percentages were reported for analyses of patient baseline characteristics, adherence rates, rates of positive mt‐sDNA test results, and rates of follow‐up colonoscopies, and *p*‐values from chi‐squared tests (or Fisher's exact tests, where applicable) were reported. For the analysis of time to adherence, the mean number of days, 95% confidence intervals (CIs), and *p*‐values from 1‐way analyses of variance (and *t*‐tests, where applicable) were reported.

Logistic regression analysis was used to assess demographics and characteristics associated with adherence to repeat screening with the mt‐sDNA test (binary outcome: yes/no). Variables were included in the model if statistically significant differences were observed in the chi‐squared tests. Final regression results were obtained from a single multivariable logistic regression model adjusted for all covariates.

All analyses were performed using R version 4.1.0 [15].

## 3 Results

**FIGURE 1:**
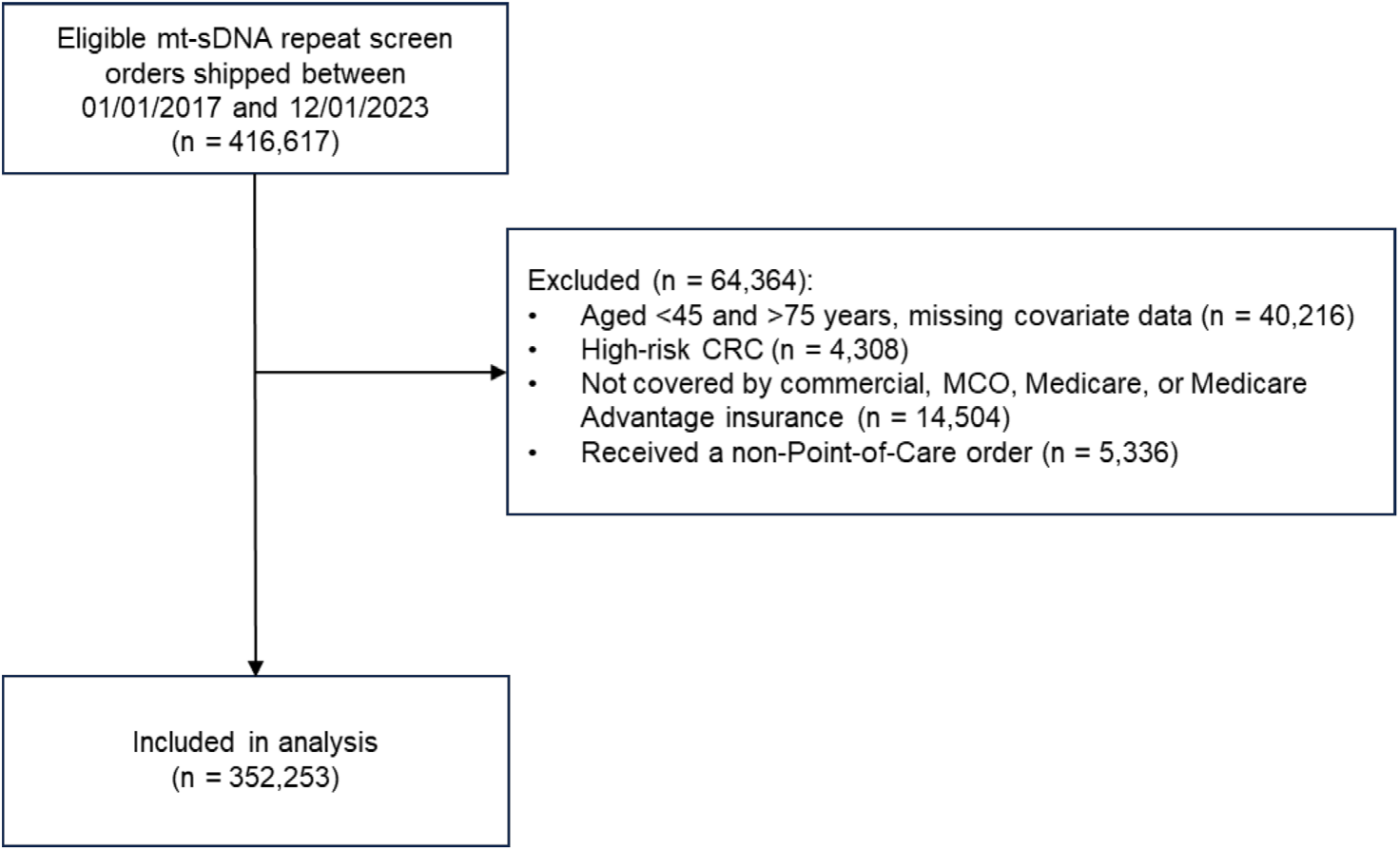
Attrition of eligible patients who continuously enrolled in a health plan with at least 1 medical claim, and 1 year before and after a multi‐target stool DNA (mt‐sDNA) repeat screen order.

Among 416,617 individuals with orders for repeat screening with the mt‐sDNA kit during the study period, 352,253 patients met the eligibility criteria and were included in the analysis (Figure 1). Most patients were female (62.1%), of White race/ethnicity (60.9%), had a CCI of 0 (76.5%), and lived in a metropolitan setting (82.9%) (Table 1). There were 63.2%, 2.5%, 7.6%, and 26.7% of patients who had commercial, MCO, Medicare, and Medicare Advantage health insurance, respectively. Most patients (71.8%) were prescribed their mt‐sDNA kit by a primary care practitioner (PCP). The majority of patients performed one earlier mt‐sDNA test (98.1%); 1.9% of patients performed two earlier tests, and 0.003% performed three earlier mt‐sDNA tests.

**TABLE 1:** Baseline demographic characteristics by type of health insurance.

*n* (%)	Overall (*N* = 352,253)	Commercial (*N* = 222,493)	MCO (*N* = 8792)	Medicare (*N* = 26,883)	Medicare advantage (*N* = 94,085)
Overall	352,253 (100.0)	222,493 (63.2)	8792 (2.5)	26,883 (7.6)	94,085 (26.7)
Age group (years)					
45–64	211,195 (60.0)	192,483 (86.5)	8143 (92.6)	1752 (6.5)	8817 (9.4)
65–75	141,058 (40.0)	30,010 (13.5)	649 (7.4)	25,131 (93.5)	85,268 (90.6)
Sex					
Female	218,561 (62.1)	135,315 (60.8)	5650 (64.3)	17,661 (65.7)	59,935 (63.7)
Male	133,692 (38.0)	87,178 (39.2)	3142 (35.7)	9222 (34.3)	34,150 (36.3)
Race/ethnicity					
Asian	9782 (2.8)	6514 (2.9)	351 (4.0)	589 (2.2)	2328 (2.5)
Black	23,506 (6.7)	12,692 (5.7)	952 (10.8)	1683 (6.3)	8179 (8.7)
Hispanic or Latino	20,415 (5.8)	13,590 (6.1)	629 (7.2)	1188 (4.4)	5008 (5.3)
White	214,614 (60.9)	112,759 (50.7)	5304 (60.3)	22,320 (83.0)	74,231 (78.9)
Other/unknown	83,936 (23.8)	76,938 (34.6)	1556 (17.7)	1103 (4.1)	4339 (4.6)
Charlson Comorbidity Index					
0	269,563 (76.5)	178,154 (80.1)	6112 (69.5)	19,318 (71.9)	65,979 (70.1)
1	64,461 (18.3)	35,716 (16.1)	2081 (23.7)	5605 (20.9)	21,059 (22.4)
2	13,329 (3.8)	6486 (2.9)	402 (4.6)	1400 (5.2)	5041 (5.4)
3	3096 (0.9)	1268 (0.6)	103 (1.2)	365 (1.4)	1360 (1.5)
4	719 (0.2)	260 (0.1)	33 (0.4)	97 (0.4)	329 (0.4)
5	200 (0.1)	77 (0.03)	7 (0.1)	24 (0.1)	92 (0.1)
> 6	885 (0.3)	532 (0.2)	54 (0.6)	74 (0.3)	225 (0.2)
Urban/rural classification					
Metropolitan	291,857 (82.9)	185,711 (83.5)	6572 (74.8)	22,208 (82.6)	77,366 (82.2)
Micropolitan	34,574 (9.8)	21,029 (9.5)	1182 (13.4)	2661 (9.9)	9702 (10.3)
Rural	10,963 (3.1)	6491 (2.9)	449 (5.1)	875 (3.3)	3148 (3.4)
Small town	14,859 (4.2)	9262 (4.2)	589 (6.7)	1139 (4.2)	3869 (4.1)
Median income by zip code					
< $25 K	231 (0.1)	109 (0.1)	31 (0.4)	16 (0.1)	75 (0.1)
$25 K–$50 K	26,350 (7.5)	14,738 (6.6)	1276 (14.5)	1548 (5.8)	8788 (9.3)
$50 K–$75 K	136,947 (38.9)	82,472 (37.1)	4337 (49.3)	9579 (35.6)	40,559 (43.1)
$75 K–$100 K	99,107 (28.1)	63,322 (28.5)	1957 (22.3)	7810 (29.1)	26,018 (27.7)
$100 K–$200 K	84,239 (23.9)	57,970 (26.1)	1105 (12.6)	7474 (28.0)	17,690 (19.0)
> $200 K	2776 (0.8)	2251 (1.0)	19 (0.2)	223 (0.8)	283 (0.3)
Unknown	2603 (0.7)	1631 (0.7)	67 (0.8)	233 (0.9)	672 (0.7)
Provider specialty					
GI	3870 (1.1)	2012 (0.9)	127 (1.4)	561 (2.1)	1170 (1.2)
NP/PA	71,371 (20.3)	47,321 (21.3)	2511 (28.6)	4098 (15.2)	17,441 (18.5)
OB/GYN	9837 (2.8)	8429 (3.8)	159 (1.8)	325 (1.2)	924 (1.0)
PCP	252,902 (71.8)	155,339 (69.8)	5524 (62.8)	20,905 (77.8)	71,134 (75.6)
Other	14,273 (4.1)	9392 (4.2)	471 (5.4)	994 (3.7)	3416 (3.6)
Digital outreach					
No digital	52,916 (15.0)	26,795 (12.0)	1435 (16.3)	5620 (20.9)	19,066 (20.3)
Digital	276,227 (78.4)	182,455 (82.0)	6937 (78.9)	18,697 (69.6)	68,138 (72.4)
Unknown	23,110 (6.6)	13,243 (6.0)	420 (4.8)	2566 (9.6)	6881 (7.3)
Earlier mt‐sDNA tests					
1	345,657 (98.1)	218,963 (98.4)	8692 (98.9)	26,146 (97.3)	91,856 (97.6)
2	6585 (1.9)	3525 (1.6)	100 (1.1)	735 (2.7)	2225 (2.4)
3	11 (0.003)	5 (0.02)	0 (0)	2 (0.007)	4 (0.004)

Abbreviations: GI, gastroenterologist; MCO, managed care organization; mt‐sDNA, multi‐target stool DNA; NP/PA, nurse practitioner/physician assistant; OB/GYN, obstetrician/gynecologist; PCP, primary care practitioner.

### 3.1 Adherence to mt‐sDNA Testing

The adherence rate for repeat screening with the mt‐sDNA test in the overall cohort was 86.2% (*n* = 303,580; Table 2). Adherence rates according to health insurance were 85.3%, 81.1%, 91.5%, and 87.2% for commercial, MCO, Medicare, and Medicare Advantage, respectively. Relatively higher adherence rates were observed in patients aged 65–75 years (87.3%), those with a CCI of 0 (86.9%) or 1 (84.0%), and patients with median income of > $200,000 (89.2%). Adherence rates were lower for patients with Hispanic or Latino (82.8%) and Black (83.5%) race/ethnicity, compared with patients who were Asian (86.1%) or White (86.7%). Adherence rates for patients who had taken one, two, or three earlier mt‐sDNA tests were 86.1%, 90.7%, and 81.8%, respectively.

**TABLE 2:** Adherence numbers and rates by type of health insurance.

*n* (%^a^) adherent; *p* ^b^	Overall (*N* = 352,253)	Commercial (*N* = 222,493)	MCO (*N* = 8792)	Medicare (*N* = 26,883)	Medicare advantage (*N* = 94,085)
Overall	303,580 (86.2)	189,820 (85.3)	7127 (81.1)	24,610 (91.5)	82,023 (87.2)
Age group (years)	*p* < 0.0001	*p* < 0.0001	*p* = 0.0006	*p* < 0.0001	*p* < 0.0001
45–64	180,446 (85.4)	165,323 (85.9)	6624 (81.4)	1394 (79.6)	7105 (80.6)
65–75	123,134 (87.3)	24,497 (81.6)	503 (77.5)	23,216 (92.4)	74,918 (87.9)
Sex	*p* = 0.1790	*p* = 0.3111	*p* = 0.4095	*p* = 0.9178	*p* = 0.0201
Female	188,227 (86.1)	115,361 (85.3)	4565 (80.8)	16,165 (91.5)	52,136 (87.0)
Male	115,353 (86.3)	74,459 (85.4)	2562 (81.5)	8445 (91.6)	29,887 (87.5)
Race/ethnicity	*p* < 0.0001	*p* < 0.0001	*p* = 0.0119	*p* < 0.0001	*p* < 0.0001
Asian	8417 (86.1)	5582 (85.7)	282 (80.3)	527 (89.5)	2026 (87.0)
Black	19,617 (83.5)	10,455 (82.4)	741 (77.8)	1488 (88.4)	6933 (84.8)
Hispanic or Latino	16,902 (82.8)	11,214 (82.5)	491 (78.1)	1034 (87.0)	4163 (83.1)
White	186,079 (86.7)	96,066 (85.2)	4342 (81.9)	20,563 (92.1)	65,108 (87.7)
Other/unknown	72,565 (86.5)	66,503 (86.4)	1271 (81.7)	998 (90.5)	3793 (87.4)
Charlson Comorbidity Index	*p* < 0.0001	*p* < 0.0001	*p* = 0.1110	*p* < 0.0001	*p* < 0.0001
0	234,357 (86.9)	153,316 (86.1)	5005 (81.9)	17,835 (92.3)	58,201 (88.2)
1	54,130 (84.0)	29,525 (82.7)	1653 (79.4)	5048 (90.1)	17,904 (85.0)
2	11,167 (83.8)	5313 (81.9)	316 (78.6)	1250 (89.3)	4288 (85.1)
3	2492 (80.5)	989 (78.0)	78 (75.7)	316 (86.6)	1109 (81.5)
4	551 (76.6)	180 (69.2)	26 (78.8)	81 (83.5)	264 (80.2)
5	145 (72.5)	53 (68.8)	5 (71.4)	20 (83.3)	67 (72.8)
> 6	738 (83.4)	444 (83.5)	44 (81.5)	60 (81.1)	190 (84.4)
Urban/rural classification	*p* < 0.0001	*p* = 0.0081	*p* = 0.3006	*p* = 0.5583	*p* < 0.0001
Metropolitan	251,193 (86.1)	158,231 (85.2)	5329 (81.1)	20,324 (91.5)	67,309 (87.0)
Micropolitan	29,868 (86.4)	18,036 (85.8)	940 (79.5)	2451 (92.1)	8441 (87.0)
Rural	9566 (87.3)	5583 (86.0)	369 (82.2)	793 (90.6)	2821 (89.6)
Small town	12,953 (87.2)	7970 (86.1)	489 (83.0)	1042 (91.5)	3452 (89.2)
Median income by zip code	*p* < 0.0001	*p* < 0.0001	*p* < 0.0001	*p* < 0.0001	*p* < 0.0001
< $25 K	177 (76.6)	88 (80.7)	21 (67.7)	14 (87.5)	54 (72.0)
$25 K–$50 K	21,765 (82.6)	12,098 (82.1)	986 (77.3)	1367 (88.3)	7314 (83.2)
$50 K–$75 K	117,325 (85.7)	69,876 (84.7)	3477 (80.2)	8726 (91.1)	35,246 (86.9)
$75 K–$100 K	86,001 (86.8)	54,301 (85.8)	1627 (83.1)	7169 (91.8)	22,904 (88.0)
$100 K–$200 K	73,642 (87.4)	50,114 (86.5)	938 (84.9)	6910 (92.5)	15,680 (88.6)
> $200 K	2476 (89.2)	1992 (88.5)	17 (89.5)	209 (93.7)	258 (91.2)
Unknown					
Provider specialty	*p* < 0.0001	*p* < 0.0001	*p* = 0.4600	*p* = 0.0005	*p* < 0.0001
GI	3340 (86.3)	1697 (84.3)	101 (79.5)	497 (88.6)	1045 (89.3)
NP/PA	60,879 (85.3)	40,075 (84.7)	2034 (81.0)	3711 (90.6)	15,059 (86.3)
PCP	218,521 (86.4)	132,676 (85.4)	4496 (81.4)	19,207 (91.9)	62,142 (87.4)
OB/GYN	8599 (87.4)	7336 (87.0)	129 (81.1)	303 (93.2)	831 (89.9)
Other	12,241 (85.8)	8036 (85.6)	367 (77.9)	892 (89.7)	2946 (86.2)
Digital outreach	*p* < 0.0001	*p* < 0.0001	*p* = 0.0055	*p* = 0.0002	*p* < 0.0001
No digital	44,247 (83.6)	21,898 (81.7)	1130 (78.8)	5088 (90.5)	16,131 (84.6)
Digital	240,408 (87.0)	157,874 (86.5)	5671 (81.8)	17,202 (92.0)	59,661 (87.6)
Unknown	18,925 (81.9)	10,048 (75.9)	326 (77.6)	2320 (90.4)	6231 (90.6)
Earlier mt‐sDNA tests	*p* < 0.0001	*p* < 0.0001	*p* = 0.7121	*p* = 0.0004	*p* < 0.0001
1	297,600 (86.1)	186,671 (85.3)	7044 (81.0)	23,906 (91.4)	79,979 (87.1)
2	5971 (90.7)	3145 (89.2)	83 (83.0)	702 (95.5)	2041 (91.7)
3	9 (81.8)	4 (80.0)	0 (0)	2 (100.0)	3 (75.0)

Abbreviations: GI, gastroenterologist; MCO, managed care organization; mt‐sDNA, multi‐target stool DNA; NP/PA, nurse practitioner/physician assistant; OB/GYN, obstetrician/gynecologist; PCP, primary care practitioner.

^a^ Adherence rates reflect the percentage of patients who adhered to repeat screening within respective demographic subgroups among the overall cohort or by health insurance plan.

^b^ *p*‐values are based on chi‐squared tests by category.

**FIGURE 2:**
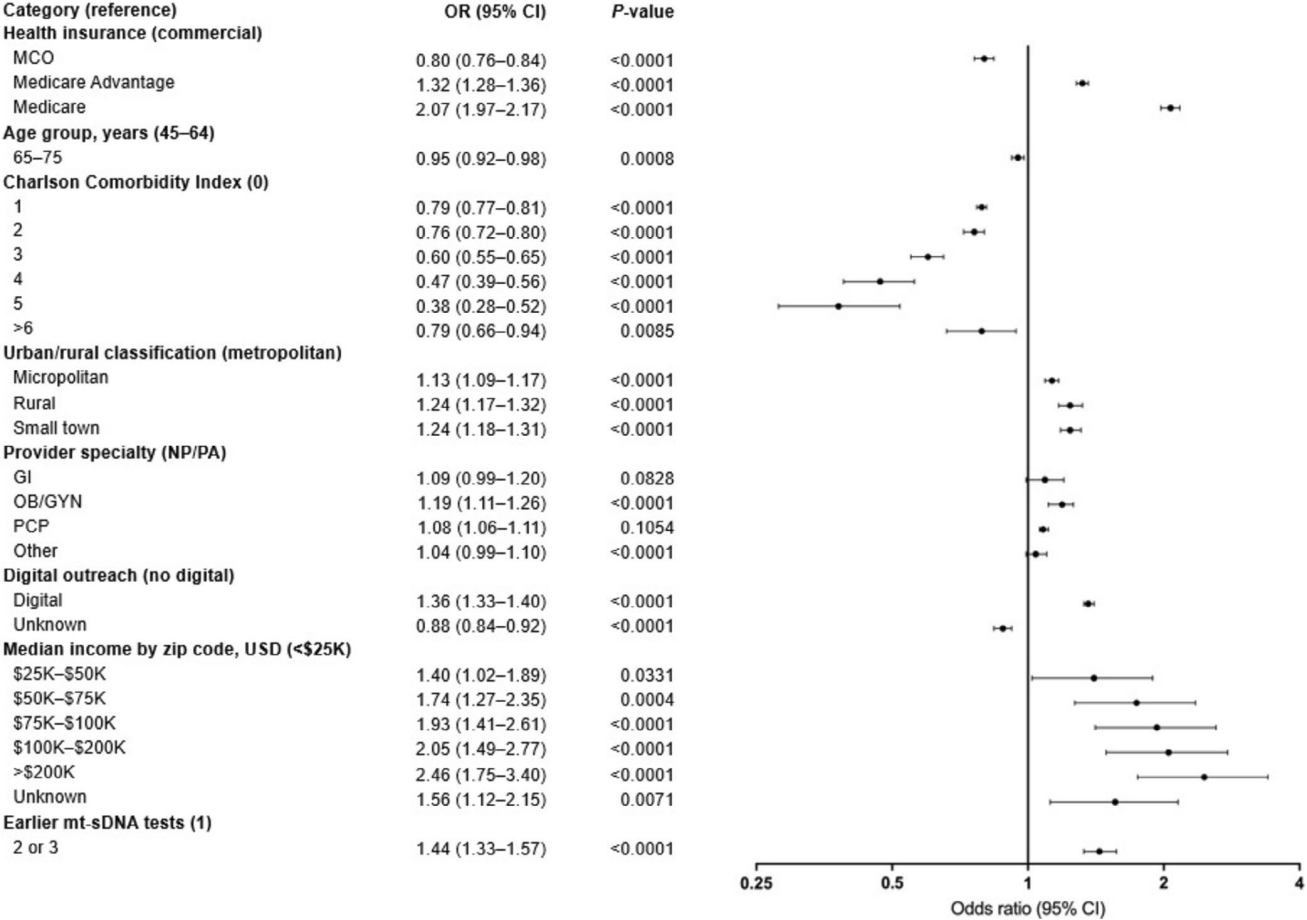
Logistic regression for factors associated with adherence among adherent patients (*N* = 303,580). The *x*‐axis is depicted on a log base 2 scale. CI, confidence interval; GI, gastroenterologist; MCO, managed care organization; mt‐sDNA, multi‐target stool DNA; NP/PA, nurse practitioner/physician assistant; OB/GYN, obstetrician/gynecologist; OR, odds ratio; PCP, primary care practitioner.

In the logistic regression analysis, age group, number of comorbidities, urban/rural setting, median household income, type of insurance coverage, provider specialty, receipt of digital outreach, and number of earlier mt‐sDNA tests were strong predictors of adherence to repeat screening with the mt‐sDNA kit (Figure 2). Age 65–75 years was associated with slightly lower adherence to repeat screening (odds ratio [OR] 0.95; 95% CI 0.92–0.98), compared with age 45–64 years. Patients with multiple comorbidities (CCI of ≥ 1) were less likely to adhere to repeat screening compared with those without comorbidities (CCI of 0), while individuals from micropolitan, rural, or small‐town settings were more likely to adhere to repeat screening than those from metropolitan areas. Compared with a median household income of < $25,000, higher household income was associated with higher adherence to repeat screening, particularly a median income of $100,000–$200,000 (OR 2.05; 95% CI 1.49–2.77) or > $200,000 (OR 2.46; 95% CI 1.75–3.40).

Patients with MCO insurance were less likely to adhere to repeat screening (OR 0.80; 95% CI 0.76–0.84), while enrollment in Medicare Advantage (OR 1.32; 95% CI 1.28–1.36) or Medicare (OR 2.07; 95% CI 1.97–2.17) was associated with higher adherence, compared with commercial health insurance (Figure 2). Among provider specialties, patients were more likely to adhere to repeat screening when the mt‐sDNA test was prescribed by an obstetrician or gynecologist (OR 1.19; 95% CI 1.11–1.26) or a PCP (OR 1.08; 95% CI 1.06–1.11), compared with a nurse practitioner or physician assistant. Individuals who received digital outreach were more likely to adhere to repeat screening compared with those who did not receive digital outreach (OR 1.36; 95% CI 1.33–1.40). Patients who underwent at least two earlier mt‐sDNA tests were more likely to adhere to repeat screening compared with those who had taken one earlier mt‐sDNA test (OR 1.44; 95% CI 1.32–1.57).

### 3.2 Time to Adherence

The mean (95% CI) time to a successful mt‐sDNA result was 35.6 (35.4–35.7) days in the overall cohort. Mean (95% CI) time to a successful mt‐sDNA result according to health insurance type was 32.0 (31.7–32.3), 31.4 (31.0–31.9), 37.6 (37.4–37.8), and 37.3 (36.2–38.5) days for commercial, MCO, Medicare, and Medicare Advantage, respectively (*p* < 0.0001). Mean (95% CI) time to a successful mt‐sDNA result for patients who had taken one, two, or three earlier mt‐sDNA tests was 35.7 (35.5–35.8), 30.7 (29.7, 31.7), and 33.0 (14.6, 51.4) days, respectively (*p* < 0.0001).

### 3.3 Positive Test Result and Colonoscopy Rates

Among patients who had a successful mt‐sDNA test (*n* = 303,691), 11.9% (*n* = 36,063) had a positive result (Table 3). Of patients with a positive mt‐sDNA test result, most underwent colonoscopy (*n* = 27,334, 75.8%). Rates of colonoscopy were lower for individuals of Asian (73.8%), Black (69.8%), and Hispanic/Latino (73.2%) race or ethnicity, compared with individuals who were White (75.5%). Colonoscopy rates generally increased with median household income, ranging from 76.2% for median income < $25,000 to 70.6% for $25,000–$50,000 to 86.3% for > $200,000. Colonoscopy rates were lower for patients with MCO insurance (65.0%) and those enrolled in Medicare Advantage (70.0%), compared with patients enrolled in Medicare (77.5%) or had commercial insurance (80.1%). Among patients who had taken one, two, or three earlier mt‐sDNA tests and had a positive mt‐sDNA result, similar proportions of patients underwent colonoscopy (*p* = 0.2306).

**TABLE 3:** Positive test results and follow‐up colonoscopies among patients who returned the mt‐sDNA test (*N* = 303,691).

*n* (%); *p* ^a^	mt‐sDNA test‐positive^b^	Colonoscopy^c^
Overall	36,063 (11.9)	27,334 (75.8)
Age group (years)	*p* < 0.0001	*p* < 0.0001
45–64	17,236 (9.6)	13,556 (78.7)
65–75	18,827 (15.3)	13,778 (73.2)
Sex	*p* < 0.0001	*p* = 0.01626
Female	21,075 (11.2)	15,877 (75.3)
Male	14,988 (13.0)	11,457 (76.4)
Race/ethnicity	*p* < 0.0001	*p* < 0.0001
Asian	680 (8.1)	502 (73.8)
Black	2129 (10.9)	1485 (69.8)
Hispanic or Latino	1636 (9.7)	1197 (73.2)
White	24,658 (13.3)	18,607 (75.5)
Other/unknown	6960 (9.6)	5543 (79.6)
Charlson Comorbidity Index	*p* < 0.0001	*p* = 0.0861
0	25,739 (11.0)	19,579 (76.1)
1	7624 (14.1)	5718 (75.0)
2	1927 (17.3)	1474 (76.5)
3	521 (20.9)	370 (71.0)
4	138 (25.0)	107 (77.5)
5	41 (28.3)	30 (73.2)
> 6	73 (9.9)	56 (76.7)
Urban/rural classification	*p* < 0.0001	*p* = 0.0909
Metropolitan	29,349 (11.7)	22,184 (75.6)
Micropolitan	3789 (12.7)	2878 (76.0)
Rural	1257 (13.1)	973 (77.4)
Small town	1668 (12.9)	1299 (77.9)
Median income by zip code	*p* < 0.0001	*p* < 0.0001
< $25 K	21 (11.9)	16 (76.2)
$25 K–$50 K	2805 (12.9)	1981 (70.6)
$50 K–$75 K	14,851 (12.7)	11,052 (74.4)
$75 K–$100 K	10,138 (11.8)	7782 (76.8)
$100 K–$200 K	7753 (10.5)	6122 (79.0)
> $200 K	226 (9.1)	195 (86.3)
Unknown	269 (12.3)	186 (69.1)
Health insurance	*p* < 0.0001	*p* < 0.0001
Commercial	18,396 (9.7)	14,726 (80.1)
MCO	920 (12.9)	598 (65.0)
Medicare Advantage	12,967 (15.8)	9082 (70.0)
Medicare	3780 (15.4)	2928 (77.5)
Provider specialty	*p* < 0.0001	*p* = 0.0003
GI	464 (13.9)	369 (79.5)
NP/PA	7346 (12.1)	5476 (74.5)
OB/GYN	752 (8.7)	608 (80.9)
PCP	26,090 (11.9)	19,797 (75.9)
Other	1411 (11.5)	1084 (76.8)
Digital outreach	*p* < 0.0001	*p* = 0.0025
No digital	5678 (12.8)	4247 (74.8)
Digital	27,880 (11.6)	21,243 (76.2)
Unknown	2505 (13.2)	1844 (73.6)
Earlier mt‐sDNA tests	*p* = 0.2412	*p* = 0.23055
1	35,311 (11.9)	26,745 (75.7)
2	751 (12.6)	588 (78.3)
3	1 (11.1)	1 (100.0)

Abbreviations: CRC, colorectal cancer; GI, gastroenterologist; MCO, managed care organization; mt‐sDNA, multi‐target stool DNA; NP/PA, nurse practitioner/physician assistant; OB/GYN, obstetrician/gynecologist; PCP, primary care practitioner.

^a^ *p*‐values are based on chi‐squared tests by category.

^b^ Percentages calculated among individuals with a successful mt‐sDNA result on or before 365 days (*N* = 303,691) in the respective subgroup.

^c^ Percentages calculated among mt‐sDNA‐positive individuals in the respective subgroup.

## 4 Discussion

The current study evaluated adherence to repeat CRC screening using the mt‐sDNA kit among insured US patients aged 45–75 years with average risk for CRC, informed by high‐quality data linked to medical claims. With more than 350,000 insured US adults included, this is one of the largest real‐world evaluations of repeat CRC screening behavior using the mt‐sDNA test conducted to date. The diverse mix of health insurance types supports the generalizability of the study results across different insured populations within the US healthcare system.

Among a cohort of patients who had previously completed at least one mt‐DNA screening test, overall adherence to repeat CRC screening with mt‐sDNA was high at 86.2%. Of patients with a positive test result, the majority (75.8%) underwent follow‐up colonoscopy.

The present study included detailed subgroup analyses across demographic, urban/rural classification, and socioeconomic strata, which can provide valuable insights into potential health disparities. Although there were differences in adherence between subgroups, adherence rates were > 80% for most of the demographic subgroups evaluated; these include populations who historically have been demonstrated to have lower screening rates, such as patients of Black race (83.5%), patients with Hispanic ethnicity (82.8%), and those who live in rural (87.3%) or small‐town settings (87.2%) [4, 16, 17]. Consistent with other reports, individuals with multiple comorbidities in our study (particularly those with CCI of 4 or 5) and those with lower median household income were less likely to be adherent [4, 17, 18]. Patients with CCI scores > 6 were more likely to be adherent than patients with CCI scores of 4–5, which may reflect increased healthcare engagement among patients with more complex medical needs.

Barriers to CRC screening adherence are well documented; these include a lack of awareness, cost factors, and a lack of physician recommendation [5, 19, 20]. A study of patient‐reported CRC screening barriers among US individuals at average risk found that patients were concerned about pain or discomfort from more invasive screening tests, and they had a fear of the procedures in general [19]. Indeed, adherence rates are dependent on the type of screening modality, and less invasive tests are generally expected to have higher uptake [5]. Nonetheless, adherence to repeat screening with FOBT (including FIT) is reportedly low, ranging from 10.6% to 38.8% [21, 22]. A systematic review of studies evaluating repeat FOBT testing found that, among patients completing a first round of FOBT, median rates of repeat testing were 82.0% in round 2 [23]. A median of 46.6% of patients completed FOBT in two consecutive rounds [23]. In studies examining up to three rounds of FOBT testing, a median of 39.2% of patients completed repeat FOBT testing [23].

Adherence rates in our current study were > 82% in individuals who underwent one to three earlier mt‐sDNA tests. Furthermore, regression analyses showed that, in contrast to the findings of the aforementioned FOBT systematic review, patients who underwent two or three earlier mt‐sDNA tests were more likely to adhere to repeat screenings compared with patients who had one earlier mt‐sDNA test (OR 1.44). This indicates that patients who have used mt‐sDNA tests in the past are likely to use them again in the future. Furthermore, patients who chose to receive the enhanced navigation and support system via digital outreach were more likely to adhere to repeat mt‐sDNA screening than those who did not (OR 1.37); this finding is particularly relevant given the current widespread availability and adoption of digital communication tools. It is also possible that individuals with an affinity for digital communication tools are more likely to adhere to recommended testing.

The observed high adherence may be attributed to a number of factors, including the simplicity of using the mt‐sDNA kit and the provision of patient navigation and support (including the option of receiving digital outreach). Additional factors may include brand recognition and consumer awareness, driven by national advertising campaigns and media exposure, compared with non‐branded screening options such as FIT or FOBT. While our study was not designed to isolate the effect of marketing or brand familiarity, we acknowledge that these external factors likely play a role in patient behavior and screening follow‐through. It should be noted that individuals who adhere to repeat mt‐sDNA screening may be those who are already more engaged in their healthcare in general.

Following a positive result from stool‐based screening tests, a timely colonoscopy is required for a definitive diagnosis of CRC [6], and a longer delay to colonoscopy after positive stool‐based testing is associated with increased CRC incidence, more advanced CRC stage at diagnosis, and higher CRC‐related mortality [24]. The colonoscopy rate among patients with a positive mt‐sDNA test in the current study was 75.8%, which is notably higher than the rate reported in a study of Mohl and colleagues, where only 56.1% of US patients with a positive stool‐based test result received a follow‐up colonoscopy within a year [25]. Notably, only 41.5% of the patients evaluated by Mohl and colleagues received the mt‐sDNA test, and authors further found that the rate of follow‐up colonoscopy among mt‐sDNA recipients (66.6% after 360 days) was much higher than among FIT recipients (48.7%) [25]. Hence, our findings add to the current evidence base and underscore the clinical utility of the mt‐sDNA pathway in facilitating appropriate diagnostic follow‐up. High repeat adherence observed in our study likely reflects both patient self‐selection and broader factors influencing engagement, including previous positive screening experiences, ongoing provider interaction, and possibly the test characteristics.

This study is subject to several limitations. The study population consisted exclusively of insured individuals with continuous health plan enrollment; this likely results in an overestimation of adherence compared with uninsured or underinsured individuals. Inherent in studies of claims data, other patient demographics that might affect screening adherence, such as preferred language or access to care, could not be assessed. In addition, as claims data are collected primarily for billing purposes, they may lack the clinical depth needed to fully capture factors such as symptoms and family history. Codes on diagnosis and outcomes in the dataset could have been subject to misclassification due to incomplete or inaccurate coding. Family history of cancer could be underreported and could lead to some patients being misclassified as having an average risk of CRC. Certain factors may not have been accounted for due to the retrospective nature of the analysis. Patient numbers in some subgroups were small—for example, patients with CCI of 4–6 and patients who received three earlier mt‐sDNA tests; results from these subgroup analyses should be interpreted with caution. Variables such as age, insurance type, race/ethnicity, comorbidity burden (CCI), and household income may be correlated, given their interrelationships in real‐world data, and multicollinearity may have affected the results of the regression analysis. Follow‐up procedures, such as colonoscopy, may be underreported if they were performed outside the insurance network or in settings not captured by the database; this may potentially lead to underestimation of downstream adherence. Because the current cohort included only those who had previously completed an mt‐sDNA test and remained continuously enrolled in a health plan, there is a risk of selection bias towards a more health‐engaged and health‐literate population, which may not represent the average‐risk population at large. Our study is also limited by its inability to address initial screening uptake, which could have a strong impact on screening adherence and participation in repeat screenings. The grouping of patients by ZIP code level median incomes could mask significant intra‐cluster variation and lead to false inferences.

Future work could utilize cluster‐robust standard errors or hierarchical modeling to further interrogate the impact of household income on adherence. We would also like to distinguish between patients with limited and more active healthcare engagement to determine how this may impact initial uptake of screening tests. Further, incorporating theoretical frameworks and statistical planning in future work could strengthen interpretability and provide additional structure for analyzing behavioral predictors of adherence. That said, the current study had a number of strengths, including the use of 2 large national databases.

## 5 Conclusions

In this large retrospective study of average‐risk individuals aged 45–75 years with a health insurance plan in the United States, the overall rate of persistent adherence to the mt‐sDNA test was 86.2%, and high rates of follow‐up colonoscopy (75.8%) were observed among individuals with a positive result. These rates are higher than those historically observed for other stool‐based CRC screening tests. Current findings support the use of the mt‐sDNA test in improving adherence to repeat screening among individuals at average risk for CRC. Such information may be useful to inform national strategies to enhance CRC screening uptake and reduce the burden of CRC in the United States.

## Author Contributions

**Mallik Greene:** conceptualization, methodology, validation, formal analysis, investigation, resources, data curation, writing – review and editing, visualization, supervision, project administration, funding acquisition. **Joseph Anderson:** conceptualization, methodology, interpretation, review and editing, and visualization. **Joseph LeMaster:** conceptualization, methodology, interpretation, review and editing, and visualization. **Jeffrey Arroyo:** conceptualization, methodology, interpretation, review and editing, and visualization. **Jorge Zapatier:** conceptualization, methodology, interpretation, review and editing, and visualization. **Jemel Bingham:** conceptualization, methodology, interpretation, review and editing, and visualization. **Raja Kakuturu:** oversight of data generation, data acquisition, data accuracy, interpretation, review and editing. **Jordan J. Karlitz:** conceptualization, methodology, interpretation, review and editing, and visualization. **Quang Le:** methodology, validation, formal analysis, investigation, data curation, visualization, review and editing.

## Ethics Statement

The study was considered exempt research under 45 CFR § 46.104(d)(4) as it involved only the secondary use of data that were de‐identified in compliance with the US Health Insurance Portability and Accountability Act of 1996, specifically, 45 CFR § 164.514.

## Conflicts of Interest

Mallik Greene, Raja Kakuturu, Jordan J. Karlitz, and Quang Le are employees of Exact Sciences Corporation and own stock/stock options. The other authors declare no conflicts of interest.

## Data Availability

The data that support the findings of this study are available from Exact Sciences Laboratories LLC. Restrictions apply to the availability of these data, which were used under license for this study. Data are available from the authors with the permission of Exact Sciences Laboratories LLC.
